# Developmental exposure to the pesticide malathion enhances expression of Prdm12, a regulator of nociceptor development, in *Xenopus laevis*

**DOI:** 10.17912/micropub.biology.000786

**Published:** 2023-03-20

**Authors:** Valeria Donoso, Jeremy Whitson, Barbara Lom

**Affiliations:** 1 Biology, Davidson College, Davidson, North Carolina, United States; 2 Preventative Medicine, Northwestern University, Chicago, Illinois, United States; 3 Biology, High Point University, High Point, North Carolina, United States

## Abstract

The transcription factor Prdm12 exerts important influences on the development of nociceptors, peripheral touch and pain-sensing neurons, and has been implicated in human pain sensation disorders. We examined the consequences of exposing developing *Xenopus laevis* embryos to the commonly used pesticide malathion on Prdm12 expression. Using qPCR and western blot analysis we observed that malathion treatment for the first six days of tadpole development significantly increased both *prdm12* mRNA levels and Prdm12 protein levels compared to controls. Consequently, early exposure to this pesticide has potential to alter nociceptor development.

**Figure 1. The pesticide malathion enhances prdm12 mRNA and Prdm12 protein expression:**
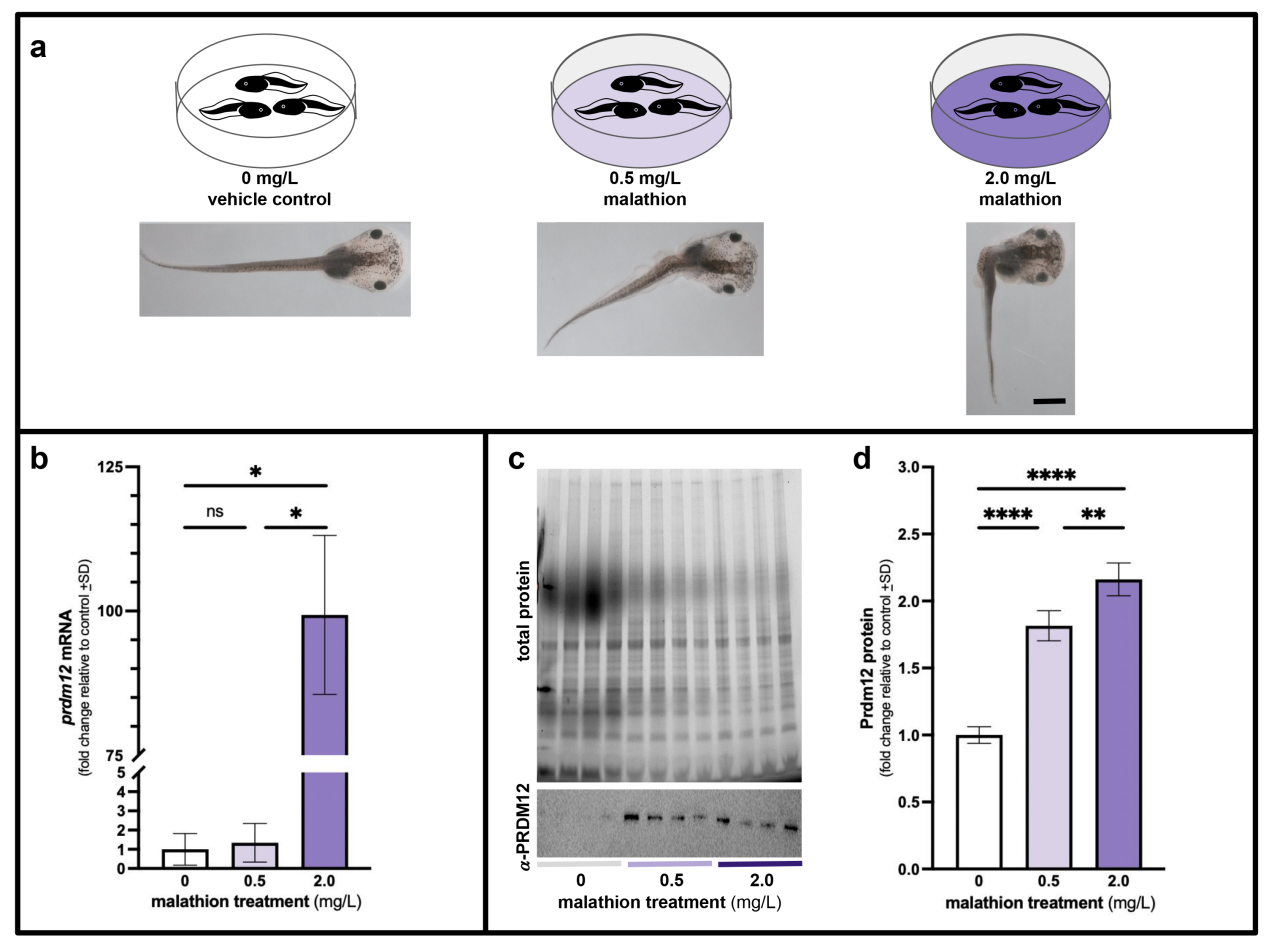
Tadpoles were raised in two concentrations of the pesticide malathion (0.5 and 2.0 mg/L) for six days until stage 47 (a) then *prdm12* mRNA (b) and Prdm12 protein (c,d) levels were assessed in comparison to vehicle controls (0 mg/L). Both concentrations of the pesticide caused noticeable gross morphological alterations in tadpole axes with more severely bent phenotypes observed in the higher concentration (a). Malathion at 0.5 mg/L did not significantly alter *prdm12* mRNA expression levels as assessed by qPCR though 2.0 mg/L malathion caused a significant 99-fold increase in *prdm12* mRNA expression levels (b). Both concentrations of malathion significantly increased Prdm12 protein levels (d) as assessed by western blot with anti-PRDM12 (c, lower panel) as normalized to total protein per lane (c, upper panel). *p=0.01; **p=0.0026; ****p<0.0001. Scale bar = 1 mm.

## Description

The PRDM family of proteins is characterized by N-terminal PR/SET domains that facilitate methyltransferase functions and C-terminal zinc finger domains that interact with nucleic acids to function in numerous aspects of development and disease (Fog et al. 2012; Hohenauer and Moore 2012; Di Zazzo et al. 2013; Di Tullio et al. 2022). Of the 19 members of the PRDM family, only PRDM12 is known to regulate the development of nociceptors, somatosensory neurons that detect and transmit touch pain sensations (Dubin and Patapoutian 2010; Rienzo et al. 2021). Developing nociceptors express the transcription factor PRDM12 and when PRDM12 expression is reduced or eliminated somatosensory neuron development and function are compromised (Kinameri et al. 2008; Bartesaghi et al. 2019; Desiderio et al. 2019; Kokotovic et al. 2021; Landy et al. 2021; Latragna et al. 2021). Transcripts of *prdm12* are detected early in *Xenopus* development, with loss-of-function experiments causing reductions in sensory neuron markers (Eguchi et al. 2015; Matsukawa et al. 2015; Nagy et al. 2015; Thélie et al. 2015; Rahman et al. 2021). Upstream, Prdm12 expression is regulated by retinoic acid and the transcription factor Zic1, with Prdm12 regulating the downstream expression of genes that influence neuronal differentiation including the neurotrophin receptor trkA, which conveys critical signals for nociceptor maturation (Jaurena et al. 2015; Desiderio et al. 2019; Baba et al. 2022).

In humans, atypical PRDM12 sequences are associated with chronic insensitivity to pain (CIP), a condition characterized by absence of pain perception caused by missing or malfunctioning nociceptors, which often results in serious injuries (Zhang et al. 2016; Moss et al. 2018; Drissi et al. 2020; Imhof et al. 2020; Kaur et al. 2020; Mehmood et al. 2020). Missense mutations in *prdm12* alter highly conserved protein residues without altering subcellular localization, while polyalanine expansion mutations cause aggregation of PRDM12, increasing instability and vulnerability to proteolysis (Chen et al. 2015). Specifically, PRDM12 recruits the methyltransferase G9a to dimethylate histone H3 at lysine 9 (H3K9me2), a function that is impaired in individuals with *prdm12* missense mutations (Yang and Shinkai 2013; Chen et al. 2015).

The organophosphate insecticide malathion is used commonly in agriculture, is applied to bodies of water to reduce mosquito populations, and is the active ingredient in some topical treatments for treating human lice infestations. Functionally, malathion impairs the ability of the synaptic enzyme acetylcholinesterase (AChE) to break down the neurotransmitter acetylcholine (ACh) within the synaptic clefts of neuromuscular junctions and can lead to detrimental outcomes such as respiratory distress, coma, muscle pain, distal numbness, delayed polyneuropathy, and death (van Helden and Bueters 1999; Krstic et al. 2008). Organophosphate-induced delayed polyneuropathy (OPIDP) includes somatosensory symptoms of degeneration of peripheral and CNS axons causing muscle pain (Lotti and Moretto 2005). Malathion activates TRPA1 channels (Patapoutian et al. 2009) expressed by sensory neurons including nociceptors enhancing neuronal excitability and pain sensations in rodents (Ding et al. 2017) and induces neuronal apoptosis, interferes with the secretion of nerve growth factor (NGF), reduces neurite outgrowth, and alters embryonic morphology (Bonfanti et al. 2004; Cook et al. 2005; Chemotti et al. 2006; Pickett et al. 2017; Venkatesan et al. 2017). Consequently, this study examined how embryonic exposure to malathion affected *prdm12* mRNA and Prdm12 protein expression in developing *Xenopus laevis* .

*Prdm12* expression is first detected in *Xenopus* embryos at stage 10 as early neurogenesis is beginning, and its expression increases thereafter through at least stage 43 (Owens et al. 2016). To determine if *prdm12* is expressed at subsequent stages of development we used qPCR to assess the expression of *prdm12* as well as three reference genes at stages 43 to 49, observing consistent expression of all four genes at these stages. C _T_ values describing *prdm12* expression were similar across all seven developmental stages (43 to 49), suggesting temporally stable expression levels of this gene (C _T_ value range: 27.1 to 29.9; C _T_ value average: 28.6 + 0.9 (SD)). We then conducted qPCR assays to examine *prdm12* expression of stage 47 *Xenopus laevis tadpoles* that developed in 0, 0.5, and 2.0 mg/L malathion. Malathion-treated tadpoles displayed bent morphologies (Fig. 1a) similar to previous observations (Bonfanti et al. 2004; Chemotti et al. 2006). In these malathion-treated tadpoles *prdm12* expression was also elevated (Fig. 1b). When compared to the vehicle control (0 mg/L malathion) averaged ∆C _T_ values of tadpoles raised in 0.5 mg/mL malathion led to an nonsignificant 1.3 fold increase in *prdm12* mRNA (p = 0.998), while tadpoles raised in 2.0 mg/L malathion exhibited a significant 99.3 fold increase in *prdm12* mRNA levels (p=0.01).

Western blots revealed that malathion also significantly increased Prdm12 protein expression in stage 47 *Xenopus laevis tadpoles* that had been raised in 0, 0.5, and 2.0 mg/L malathion (Fig. 1c). Specifically, compared to vehicle controls, treatment with 0.5 mg/L malathion significantly increased Prdm12 by 1.8 fold (p<0.0001) and treatment with 2.0 mg/L malathion significantly increased Prdm12 by 2.2 fold (p<0.0001). Together these observations indicate that exposure to malathion increases *prdm12* expression at both mRNA and protein levels. Interestingly, we observed considerable difference (~76x) in *prdm12* mRNA expression levels between the two malathion doses, yet the enhanced protein expression estimated in both doses were quite similar. Future experiments should conduct *in situ* hybridization with a probe for *prdm12* mRNA at a variety of developmental stages to determine in which cells and tissues this overexpression is specifically occurring. Further, examining potential links between malathion exposure and nociceptor development could consider assessing expression levels and patterns of additional genes known to play important roles in nociceptor development and function using RT-qPCR (e.g., TRPA1, Ntrk1, dbx1, nkx6-1, and nkx6-2). Moreover, RNAseq could be used to determine downstream effects of enhanced *prdm12* gene expression on the expression of other genes in malathion-treated tadpoles and/or methylation patterns as overexpression of *prdm12* in *Xenopus* increased H3K9me2 (Chen et al. 2015). Functionally, enhanced growth, survival, and/or function of nociceptors could be hypothesized to lead to hypersensitivity to touch if additional nociceptors develop due to enhanced Prdm12 expression or that excess Prdm12 protein could aggregate and cause nociceptor deterioration or compromise function causing harmful insensitivity to pain. Thus, future behavioral experiments to determine if malathion alters tadpole responsiveness to touch or pain are recommended to determine the functional consequences of altered Prdm12 expression.

## Methods

Adult *Xenopus laevis* frogs were maintained in environments following IACUC regulations. Embryos were obtained by overnight paired breedings with superovulation encouraged by chorionic gonadotropin hormone treatment (Sive et al. 2000). Eggs were dejellied in 2% (w/v) cysteine in 20% Steinberg’s solution (Lom and Cohen-Cory 1999) and sorted manually to select appropriately developing embryos staged according to Nieuwkoop and Faber (1994). A 10 g/L malathion stock solution was prepared in acetone then diluted immediately before treatment in 20% Steinberg’s solution to 0.5 or 2 mg/L. For each experiment three groups of typically developing 15 pre-gastrula embryos at stage 8 were placed in 30 mL of 0, 0.5, or 2 mg/L malathion and maintained at room temperature (Fig. 1a). The 0 mg/L condition included 0.02% (v/v) acetone as a vehicle control to match the solvent present in the 2 mg/L malathion condition. Tadpoles developed for six days until stage 47 and were then euthanized by lethal dose anesthesia (0.5% Tricaine-S in 20% Steinberg’s solution). Euthanized tadpoles were preserved in RNAprotect tissue reagent (100 μL/tadpole) and stored at 4° C until qPCR analysis. Biological triplicates were performed for each condition.

For qPCR analysis of mRNA expression levels, RNA was extracted from tadpoles following the protocol for Qiagen’s RNeasy Mini Kit with on-column DNase treatment. Resulting RNA concentrations were determined using a NanoDrop spectrophotometer and cDNA was synthesized using Merck’s ReadyScript® cDNA Synthesis Mix. Real-time qPCR using SYBR Green was conducted with previously published primers for *prdm12* (500 nM) and the three reference genes GAPDH (250 nM), beta-actin (500 nM), and H4 (500 nM). qPCR was conducted on 50 ng cDNA/well using NEB’s Luna Universal qPCR Master Mix by an Agilent Mx3000P qPCR System. All assays were conducted with biological triplicates and technical duplicates. C _T_ values indicated the number of cycles required for the solution to reach the threshold fluorescence, using the 2 ^-∆∆C _T_^ method to determine fold changes in expression (Livak and Schmittgen 2001). ∆C _T_ was calculated by measuring the difference between the *prdm12* and the reference gene C _T_ for each condition. The ∆∆C _T_ was found by comparing the ∆C _T_ of 0.5 and 2.0 m/L samples with the vehicle control's ∆C _T_ . To calculate the gene fold change, 2 was raised to the power of -∆∆C _T_ . Three reference genes were used to identify if experimental conditions affected the reference gene expression. ∆∆C _T_ values analyzed statistically were means of the ∆∆C _T_ values generated by the use of each different reference gene. An ordinary one-way ANOVA with Tukey’s multiple comparisons test was used to compare the three experimental conditions.

For western blot analysis of Prdm12 protein expression, tadpoles were sacrificed and homogenized, then agitated for two hours at 4 °C followed by centrifugation for 20 minutes at 12,000 rpm at 4 °C. The supernatant was collected and protein concentrations quantified by a NanoDrop spectrophotometer. Protein lysates were mixed with equal volumes of BioRad’s 2X Laemmli buffer then boiled at 100 °C for five min. A stain-free protein gel was loaded with 25 μg/lane protein and run at 150 V in tris-glycine/SDS running buffer (25 mM tris base, 190 mM glycine, and 0.1% SDS; pH 8.3). For transfer, PVDF membranes were activated for one minute in methanol and rinsed with transfer buffer (25 mM tris base, 190 mM glycine; 20% methanol) at 40 V for 70 min. The membrane was blocked with blocking buffer (5% nonfat dry milk (NFDM) in 1X TBST (20 mM tris base, 150 mM NaCl and 0.1% Tween 20)) for one hour at room temperature on a rotator. Immunostaining was conducted as described by Abcam (2020) using a primary anti-PRDM12 antibody (Sigma Aldrich HPA043143) at 1:2,000 in blocking buffer, followed by rinses in TBST, a secondary goat anti-rabbit HRP antibody (ThermoFisher 32460) at 1:1,000 dilution in blocking buffer, and rinses in TBST. The membrane was incubated with SuperSignal West Pico PLUS chemiluminescent substrate one minute at room temperature. Finally, images of membranes were taken with a Bio-Rad Imager using chemiluminescence with an exposure of 40 seconds to visualize the protein bands. Protein levels were quantified using ImageJ by comparing the mean gray value produced by the protein bands in the protein gel image and the western blot membrane image for total protein normalization (TPN; Yadav and Oh 2018). An ordinary one-way ANOVA with Tukey’s multiple comparisons test was used to compare the normalized difference between the mean gray values of the protein gel and western blot between the three treatments.

## Reagents

**Table:** 

**Reagent**	**Source**	**Catalog Number/Identifier**
acetone	Fisher Chemical	A18-500
anti-PRDM12 (primary antibody)	Sigma Aldrich	HPA043143
anti-rabbit IgG (secondary antibody)	ThermoFisher	32460
chorionic gonadotropin (hCG)	Intervet and Sigma Aldrich	Chorulon and CG10
cysteine	Sigma Aldrich	C7880
filter paper	Bio-Rad	1703958
glycine	Thermo Scientific	A13816.36
Lamelli buffer (2X)	Sigma	S3401
Luna universal qPCR master mix	NEB	M3003S
malathion	Sigma Aldrich	36143
methanol	Fisher Chemical	A452-4
nonfat dry milk (NFDM)	Saco Foods	mix ‘n drink
primers for *X.* *laevis* beta-actin	Millipore Sigma (KiCqStart primer)	XLAEVIS_148231177_1
primers for *X. laevis* GADPH and PRDM12	IDT	sequences in Thélie et al. 2015
primers for *X. laevis* H4	IDT	sequences in Šindelka et al. 2006
software (graphing/statistics)	GraphPad	Prism 9.3.1
software (image analysis)	public domain	ImageJ 1.52v
protein gels (stain-free)	Bio-Rad	4568096
PVDF membrane	Bio-Rad	1620177
ReadyScript cDNA synthesis kit	Sigma Aldrich	RDRT
RNAeasy mini kit	Quiagen	74104
RNAprotect tissue reagent	Quiagen	76104
SuperSignal West Pico PLUS chemiluminescent substrate	Thermo Scientific	34577
tricaine methanesulfonate	Western Chemical	Tricaine-S
tris base	Fisher BioReagents	BP152
Tween 20	Fisher Biotech	BP337
